# The Impact of Sleeve Gastrectomy on Hyperlipidemia: A Systematic Review

**DOI:** 10.1155/2013/643530

**Published:** 2013-10-27

**Authors:** Khalid Al Khalifa, Ahmed Al Ansari, Abdul Rahim Alsayed, Claudio Violato

**Affiliations:** ^1^Department of General Surgery, Bahrain Defense Force Hospital, Off Waly Alahed Avenue, P.O. Box 28743, West Riffa, Bahrain; ^2^Medical Education and Research Unit, Department of Community Health Sciences, Faculty of Medicine, University of Calgary, Calgary, Canada

## Abstract

*Background*. Weight loss and reduction in comorbidities can be achieved by longitudinal sleeve gastrectomy (LSG). Existing evidence suggests that LSG resolves or improves hyperlipidemia in morbidly obese patients. The aim of this study was to systematically review the effect of LSG on hyperlipidemia. *Methods*. A systematic literature search was conducted from English-language studies published from 2000 to 2012 for the following databases: MEDLINE, EMBASE, CINAHL, PubMed, Clinical evidence, Scopus, Dara, Web of Sciences, TRIP, Health Technology Database, Cochrane library, and PsycINFO. *Results*. A total of 4,211 articles were identified in the initial search, and 4,185 articles were excluded based on the exclusion criteria. Twenty-six studies met the inclusion criteria for this systematic review, involving 3,591 patients. The mean preoperative body mass index (BMI) was 48 ± 7.0 kg/m^2^ (range 37.2–65.3). The mean postoperative BMI was 35 ± 5.9 kg/m^2^ (range 26.3–49). The mean percentage of excess weight loss (EWL) was 63.1% (range 37.7–84.5), with a mean followup of 19.1 months (range 6–60). The mean levels of pre and post operative cholesterol were 194.4 ± 12.3 mg/dL (range 178–213) and 181 ± 16.3 mg/dL (range 158–200), respectively. *Conclusion*. Most patients with hyperlipidemia showed improvement or resolution of lipid profiles after LSG. Based on this systematic review, LSG has a significant effect on hyperlipidemia in the form of resolution or improvement in the majority of patients.

## 1. Introduction

Laparoscopic sleeve gastrectomy (LSG) was introduced initially as a first stage of the biliopancreatic diversion with duodenal switch (BPDDS) for severely obese patients who were regarded as high risk surgical candidates [1]. Due to its greater efficiency [2], technical simplicity [3], and low complication rates [4], LSG has become more widely accepted as a definitive treatment for morbidly obese patients [5]. In LSG, the stomach is divided vertically, while removing most of the fundus of the stomach and preserving the continuity of the digestive tract [6].

 LSG leads to long-term weight loss and improvement or resolution of its associated comorbidities such as diabetes mellitus (DM), hypertension, and hyperlipidemia [7, 8]. In a recent systematic review on the effect of the LSG on co-morbidities, Sarkhosh et al. [8] reported resolution of hypertension in 58% and resolution or improvement of hypertension in 75% of patients following LSG. In another systematic review, Alamo et al. [9] reported resolution of type 2 diabetes mellitus (T2DM) in 84% of patients after LSG. To our knowledge, the effect of LSG on hyperlipidemia was not reviewed systematically. 

 Most patients with obesity present with lipid abnormalities; however, only 20% of the obese patients population are not showing classical metabolic lipid changes [10]. Patients with abdominal obesity are more likely to have atherogenic dyslipidemia than those who have increased levels of the Low density lipoprotein (LDL) [11]. Hyperlipidemia is widely recognized as one of the main co-morbidities in severe obesity. It is therefore not surprising that research and treatment are increasingly focused on lipid profiles in the drive to potentially reduce cardiovascular related-diseases [12, 13]. The aim of this study is therefore to systematically review the published data regarding the effect of LSG on resolution or improvement of hyperlipidemia in obese patients. 

## 2. Methods

### 2.1. Search Strategy

 A systematic literature search was conducted from English-language studies published from 2000 to 2012 for the following databases: MEDLINE, EMBASE, CINAHL, PubMed, Clinical Evidence, Scopus, Dara, Web of Sciences, TRIP, Health Technology Database, Cochrane library, and PsycINFO. The potential articles from the reference lists of selected articles were searched as well. “Gray literature,” including conference abstracts, registered clinical trials, and websites was searched, including the Conference Papers Index and the Online Computer Library Center (OCLC) Papers First. The following terms were used in the search: gastric sleeve, effect of gastric sleeve on hyperlipidemia, sleeve gastrectomy, and the effect of gastric sleeve on co morbidities.

### 2.2. Data Collection and Quality Assessment

Studies of any design involving LSG for obese patients with hyperlipidemia from January 2000 to December 2012 were considered. Two independent authors then assessed the studies for relevance, inclusion, and methodological quality. The studies were classified as relevant (meeting all of the inclusion criteria), possibly relevant (meeting some but not all of the inclusion criteria), and rejected (not relevant to our review and not meeting the inclusion criteria). Each article in this study was evaluated by 2 authors independently based on the title and abstract classified as relevant or possibly relevant. Any disagreements about relevance were solved by a third coder. Based on discussions among the three coders, we achieved 100% agreement on the studies to be included. 

**Figure 1:**
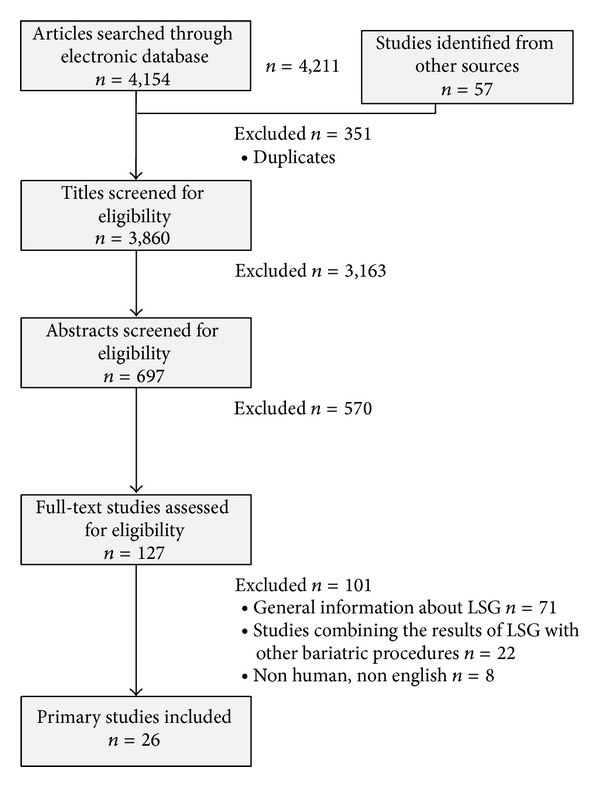
Flow chart showing systematic review search. LSG = longitudinal sleeve gastrectomy.

 The initial search yielded 4,211 articles as described in Figure 1. Of these, 351 were duplicates, 3,163 articles were excluded based on the title, further 570 articles were excluded based on the abstract, and another 101 studies were eliminated after reading the full paper. Finally, we agreed on 26 articles to be included in the present systematic review. 

### 2.3. Outcome Measures

 Studies included in this systematic review were LSG either as a single procedure or as a first procedure of staged surgery. The primary outcome of this study was resolution or improvement of hyperlipidemia. The resolution of hyperlipidemia was defined as discontinuation of all hyperlipidemia medications. The improvement of hyperlipidemia was defined as dose reduction of lipid lowering medications. The secondary outcome included changes in body mass index (BMI) postoperatively and the percentage of excess weight loss (%EWL). However, (% EWL) was calculated as follows: (% EWL) = (weight loss/excess weight) × 100, where excess weight is the total preoperative weight minus the ideal weight [14]. Ideal weight is the desirable weight which would indicate those persons with the lowest mortality rates and can be calculated through the metropolitan life table which is based on height, weight, and gender.

### 2.4. Selection Criteria

 Studies were included if they (1) were studies of any design that involved LSG, (2) included reports on the effect of LSG on lipid profile, (3) compared LSG versus any other type of bariatric surgery, (4) were studies where a second stage procedure was planned, (5) reported data on cholesterol, triglyceride, LDL, and high density lipoprotein (HDL), (6) were published in English in peer-reviewed journals, and (7) were classified as relevant or possibly relevant based on two independent authors.

 We excluded studies if (1) repeated LSG was performed, (2) they were conducted on patients who participated in health and/or nutrition program, (3) they provided only general descriptions and information about LSG without empirical data, (4) they combined LSG with another bariatric procedure results, (5) LSG was performed as revision surgery, (6) they provided information on the effect of LSG on other co-morbidities, and (7) they were nonhuman and non-English studies.

### 2.5. Assessment of Risk of Bias in Included Studies

All included studies were assessed independently by two authors for methodological quality using the Cochrane and risk of bias tools [15].

## 3. Results

### 3.1. Search Results

 As summarized in Figure 1, we agreed on 26 articles to be included in the present study. These included 18 retrospective clinical studies, 7 prospective clinical studies, and one randomized clinical trial. Five studies were published in 2012. Eight studies were published in 2011. Another seven studies were published in 2010 and two studies were published in 2008. One study was published in 2005, 2006, and 2007 (Table 1) [16–41].

### 3.2. Results of the Effects of LSG on Hyperlipidemia

 All of the 26 studies reported LSG-associated outcomes data on lipid profile-related measurements, BMI, excess weight loss and duration of followup. A total of 3,591 patients were assessed in the 26 studies. The number of patients ranged from 20 to 944. The average age of patients was 42.35 ± 5.14 years (range 30–49.5). Female represented 68.9% of the total patients. The mean preoperative BMI was 48 ± 7.0 kg/m^2^ (range 37.2–65.3). The mean postoperative BMI was 35 ± 5.9 kg/m^2^ (range 26.3–49). The mean percentage of excess weight loss was 63.1% (range 37.7–84.5), with a mean followup of 19.1 months (range 6–60) (Table 1).

 The mean levels of pre- and postoperative cholesterol were 194.4 ± 12.3 mg/dL (range 178–213), and 181 ± 16.3 mg/dL (range 158–200), respectively. The mean levels of pre- and postoperative triglyceride were 149.3 ± 21.2 mg/dL (range 120–174) and 102 ± 14.2 mg/dL (range 84–116), respectively. The mean levels of pre- and postoperative LDL were 121.3 ± 10.3 mg/dL (range 109–138) and 112 ± 3.3 mg/dL (range 109–117), respectively, and the mean levels of pre- and postoperative HDL were 46.4 ± 2.8 mg/dL (range 42–49), and 54 ± 9.3 mg/dL (range 43–64), respectively.

 Within the 26 studies included in this systematic review, 11 reported both resolution and improvement of hyperlipidemia after LSG and 83.5% of the patients had experienced resolution or improvement of hyperlipidemia. Another 7 studies reported only hyperlipidemia resolution and 54% of patients had complete resolution of hyperlipidemia. One study reported improvement of hyperlipidemia in 42% of the patients. 

 Five studies compared the lipid profile results pre and post-surgery [19, 23, 37, 39, 41]. Only three studies showed minimal changes between pre and post operative cholesterol and LDL levels [18, 19, 41]. However, the same three studies reported significant changes in triglyceride level and HDL level post LSG (Table 2).

## 4. Discussion

 The main purpose of this systematic review was to investigate further the effect of LSG on hyperlipidemia. LSG was initially used as a first stage operation for high risk surgical patients prior to undergoing a gastric bypass or biliopancreatic diversion. Existing evidence has suggested that LSG is effective as a single procedure for the treatment of morbid obesity and improvement or resolution of co-morbidities. 

 Our systematic review showed that LSG resolved or improved hyperlipidemia in a majority of patients. 83.5% of patients had resolution or improvement of their hyperlipidemia and 54% experienced complete resolution of their hyperlipidemia. These results correspond with other bariatric procedures. Omana et al. [32] found a greater resolution or improvement of hyperlipidemia with LSG in comparison with laparoscopic adjustable gastric banding. Hyperlipidemia improved in 87% of patients after LSG and in 50% of patients after gastric banding after a 15-month followup period. 

 Benaiges et al. [19] reported lower improvement or resolution rates for hyperlipidemia in the LSG group than in the laparoscopic Roux-en-Y gastric bypass (LRYGB). The hyperlipidemia improvement or resolution rate was 100% for LRYGB versus 75% for LSG. Researchers argued that the decrease in LDL cholesterol observed for LRYGB could be related to the malabsorption effect produced by this technique. This hypothesis is supported by several data [14, 42]. 

 Similarly, Kehagias et al. [28] found less resolution of hyperlipidemia in patients after LSG versus patients who underwent LRYGB. The rate of hyperlipidemia resolution was 90% for patients who had LRYGB and 75% for patients who had LSG. Lakdawala et al. [29] reported almost similar results for patients who had LSG and patients who had LRYGB. At one-year followup, hyperlipidemia resolved in 75% of patients who underwent LSG and in 78% of those who underwent LRYGB. Skroubis et al. [36] reported 90%, 78.4%, 55%, and 48.7% resolutions of hyperlipidemia after a 5-year followup for patients who underwent biliopancreatic diversion, vertical banded plasty, LSG, and Roux-en-Y gastric bypass (RYGB) respectively.

 In regard to the secondary outcome, our systematic review showed a reduction in the %EWL over a one-year followup. The percentage of EWL after LSG was 63.1% (range 37.7–84.5). In comparison with other bariatric procedures, Kehagias et al. [28] reported greater loss of the %EWL in the LSG group in the first 2 years of their study and greater in the third year but with no statistical significance. Kehagias et al. [28] went further and found that the proportion of patients who achieved a %EWL greater than 50% at 3 years postoperatively was 77% after LRYGB and 83% after LSG. 

 Omana et al. [32] reported greater loss of %EWL with LSG being 50.6% versus 40.3% with laparoscopic adjustable gastric banding. Lakdawala et al. [29] reported 50.5% loss of %EWL 6 months after LSG versus 41.7% loss of %EWL after LRYGB. 

## 5. Limitation

The primary studies included in this systematic review were case series and nonrandomized controlled trials, which are inherently biased. There were no published randomized controlled studies comparing LSG and medical therapy assessments of the resolution of hyperlipidemia on obese patients. Given this fact, the result of our systematic review should be interpreted with caution to avoid misleading results. This fact, however, appears to be applied to the majority of bariatric surgical data. Furthermore, a formal meta-analysis could not feasibly be conducted due to the high degree of heterogeneity in the study's design, intervention, and the population in the studies. With all these limitations, nevertheless, our review supports the idea that LSG is associated with the resolution and/or improvement of hyperlipidemia after LSG. 

## 6. Conclusion

 This systematic review shows that LSG has a significant effect on hyperlipidemia, producing resolution or improvement in most of the cases. Therefore, LSG remains a viable surgical option for weight loss and reduction in co-morbidities such as hyperlipidemia. 

## Figures and Tables

**Table 1:** Baseline characteristics within included studies for systematic review.

Investigator	Study design	Patient *n*	Mean age (year)	Gender (% F)	Mean BMI (kg/m^2^)	Surgery	Post-op (BMI)	Followup period (months)
Abbatini et al. [16], 2010	RCS	20	46.6	60.00%	51.6	LSG		36
Atkins et al. [17], 2012	RCS	291	45.8	76.90%	42.2	LSG		48
Benaiges et al. [18], 2011	PCS	45	44.1	78.60%	44.6	LSG	28.9	12
Benaiges et al. [19], 2012	PCS	51	44.5	82.30%	44.6	LSG	28.5	12
Boza et al. [20], 2012	RCS	773	36.9	100%	37.4	LSG	27.2	12
Cottam et al. [21], 2006	PCS	126	49.5	53.00%	65.3	LSG	49.0	12
Chowbey et al. [22], 2010	RCS	75	44.4	53.30%	58.0	LSG	37.7	12
Hady et al. [23], 2012	RCS	100	47.9	52.00%	52.2	LSG	38.0	6
D'Hondt et al. [24], 2011	RCS	83	40.4	73.50%	39.3	LSG		12
Han et al. [25], 2005	RCS	60	30.0	86.70%	37.2	LSG	28.0	12
Hutter et al. [26], 2011	RCS	944	46.5	75.00%	46.2	LSG	34.4	12
Kasama et al. [27], 2008	RCS	23	38.0	26.10%	49.1	LSG	42.1	12
Kehagias et al. [28], 2011	RCT	30	33.7	73.30%	44.9	LSG		36
Lakdawala et al. [29], 2010	RCS	50	38.0	52.00%	46.0	LSG	26.3	12
Leivonen et al. [30], 2011	PCS	55	48.5	69.00%	49.5	LSG	38.2	12
Nienhuijs et al. [31], 2010	PCS	74	42.0	60.80%	51.0	LSG	39.2	12
Omana et al. [32], 2010	RCS	49	45.0	73.50%	52.0	LSG	37.8	12
Prasad et al. [33], 2012	RCS	108	39.3	76.40%	44.5	LSG	30.2	36
Ramalingam and Anton [34], 2011	RCS	20	43.6	45.00%	42.5	LSG	33.1	12
Sammour et al. [35], 2010	PCS	100	43.0	80.00%	50.3	LSG		12
Skroubis et al. [36], 2011	RCS	151	32.8		43.1	LSG		60
Todkar et al. [37], 2010	RCS	23	44.6	73.90%	40.7	LSG	30.9	36
Weiner et al. [38], 2007	RCS	120	40.3	71.60%	60.7	LSG	38.0	12
Wong et al. [39], 2011	RCS	37	46.0	78.40%	46.0	LSG	33.0	9.2
Ou Yang et al. [40], 2008	PCS	138		64.50%	50.6	LSG	39.8	24
Strain et al. [41], 2011	RCS	45	47.3	85.00%	57.5	LSG	39.9	12

Mean/total		3591	42.3	68.90%	48.0		35.0	19.1

RCS: retrospective clinical study, LSG: laparoscopic sleeve surgery, PCS: prospective clinical study, BMI: body mass index, and RCT: randomized clinical trial.

**Table 2:** Laparoscopic sleeve gastrectomy outcomes: systematic review.

Investigator	No of patients with ↑ lipid	Cholesterol		Triglyceride		LDL		HDL		EWL %	Results (number or %)		Sta
		Pre	Post	Pre	Post	Pre	Post	Pre	Post		Res	Imp	
Abbatini et al. [16], 2010	7									68	2	26	6
Atkins et al. [17], 2012	50											42%	
Benaiges et al. [18], 2011	12	194	200	133	86	120	117	49	63	82.7	75%		
Benaiges et al. [19], 2012	51	192	196	120	84	119	115	48	64				
Boza et al. [20], 2012	112									84.5	95	11	6
Cottam et al. [21], 2006	65									46	73%	5%	
Chowbey et al. [22], 2010	40	178	158	162	101					52.3	34		
Hady et al. [23], 2012	100	213	182	166	116	138	111	42	43	49			
D'Hondt et al. [24], 2011	36									81.5	25	3	
Han et al. [25], 2005	20									83.3	65%	10%	
Hutter et al. [26], 2011	311										35%		
Kasama et al. [27], 2008	9										3	3	
Kehagias et al. [28], 2011	8									68.5	6		
Lakdawala et al. [29], 2010	50									76.1	75%		
Leivonen et al. [30], 2011	18		4.7		3		1.3		1.4	49.2	15%		3
Nienhuijs et al. [31], 2010	14									49.2	5%	4	5
Omana et al. [32], 2010	15									37.7	13	2	
Prasad et al. [33], 2012	42									66.1	86%	14%	
Ramalingam and Anton [34], 2011	4									49.6	3	1	
Sammour et al. [35], 2010	25									62.9	5		20
Skroubis et al. [36], 2011	26									52.7			
Todkar et al. [37], 2010	21	203	167	174	116	120	110	45	47	74.5			
Weiner et al. [38], 2007	34									68	2	26	6
Wong et al. [39], 2011	37	5.3	4.9	1.8	1.2	3.2	3	1.3	1.4				
Ou Yang et al. [40], 2008	23									46.1	87%		
Strain et al. [41], 2011	25	186	186	141	109	109	110	48	54				

Mean/total	1,175	194	181	149	102	121	112	47	54	63.1			

Pre: preoperatively, Post: postoperatively, LDL: low density lipoprotein, HDL: high density lipoprotein, EWL: excess weight loss, Res: resolved, Imp: improved, Sta: stable, and ↓%: decrease in lipid %.
